# Alterations in the fecal virome and bacteriome–virome interplay in IPAH

**DOI:** 10.1186/s12931-026-03797-x

**Published:** 2026-07-01

**Authors:** Yaona Bing, Wen Yuan, Lirong Liang, Jifeng Li, Yihang Chen, Lin Feng, Xuyan Li, Huilun Li, Jiuchang Zhong, Lan Wang, Zhaohui Tong, Xiaoyan Liu

**Affiliations:** 1 Medical Research Center, Beijing Institute of Respiratory Medicine and Beijing Chao-Yang Hospital, Capital Medical University, Beijing, 100020 China; 2 Heart Center, Beijing Chao-Yang Hospital, Capital Medical University, Beijing, 100020 China; 3 Evidence-Based Medicine Center, Beijing Institute of Respiratory Medicine and Beijing Chao-Yang Hospital, Capital Medical University, Beijing, China; 4 Department of Respiratory and Critical Care Medicine, Beijing Institute of Respiratory Medicine and Beijing Chao-Yang Hospital, Capital Medical University, Beijing, 100020 China; 5 Beijing Key Laboratory of Multimodal Intelligent Diagnosis and Treatment System for Respiratory Diseases, Beijing Chao-Yang Hospital, Capital Medical University, Beijing, China; 6 Department of Pulmonary Circulation, Shanghai Pulmonary Hospital Affiliated to Tongji University School of Medicine, Shanghai, China

**Keywords:** Pulmonary arterial hypertension, Gut microbiota, Gut virome

## Abstract

**Background:**

Idiopathic pulmonary arterial hypertension (IPAH) is a life-threatening cardiovascular disorder characterized by complex multisystem disturbances. Although alterations in the gut microbiota have been reported in IPAH, how the gut virome interacts with bacterial communities and host metabolism remains unclear.

**Methods:**

We enrolled 28 patients with IPAH and 30 age-matched healthy controls (HCs). Fecal viromes and bacteriomes were profiled by metagenomic sequencing, and serum metabolomic data were integrated to construct virus–bacterium–metabolite interaction networks. Random forest models were used to evaluate the diagnostic potential of virome features.

**Results:**

IPAH patients exhibited markedly reduced gut virome diversity (Shannon, Simpson, and Pielou indices, *p* < 0.05) and distinct community structures from HCs (*p* < 0.01). A total of 499 differential viral operational taxonomic units (vOTUs) were identified, accompanied by extensive reorganization of interaction networks. At the phylum level, *Hofneiviricota* was enriched and *Phixviricota* depleted, both correlating with clinical indicators. Virus–bacterium associations were markedly increased in IPAH (44,894 vs. 17,920, r > 0.5). Notably, vOTU2967, vOTU1924, and vOTU4522 were elevated and inversely related to *Bacteroides*, whose depletion was associated with increased lactic acid levels. Mediation analysis confirmed significant indirect virus–bacterium–metabolite effects (*p* < 0.05). Random forest models based on vOTUs or viral families effectively distinguished IPAH patients from controls, highlighting the exploratory potential of gut virome features for mechanistic insights.

**Conclusions:**

IPAH is characterized by reduced virome diversity, altered viral taxa, and reorganized virus–bacterium–metabolite networks. These findings suggest that gut viruses may influence disease progression by modulating bacterial metabolism, providing a potential avenue for biomarker discovery and therapeutic intervention.

**Supplementary Information:**

The online version contains supplementary material available at https://doi.org/10.1186/s12931-026-03797-x.

## Introduction

Pulmonary arterial hypertension (PAH) is a progressive and life-threatening disorder marked by sustained elevation of pulmonary vascular resistance, leading to vascular remodeling and eventual right heart failure [1]. Idiopathic PAH (IPAH), the most common sporadic form [2],, involves complex disturbances across genetic, immune, and endothelial pathways [3], and signaling pathways such as Notch3 have been shown to play a key role in vascular remodeling in PAH [4]. Although specific drug therapies have improved outcomes, long-term prognosis is still poor [5]. These limitations highlight the need to better understand its underlying mechanisms. The intestinal microbial ecosystem consists of bacteria, viruses, fungi, and other microorganisms. Growing evidence links gut microbial imbalance with cardiovascular and respiratory disorders [6–13]. In PAH, previous studies have described gut bacterial dysbiosis characterized by alterations in multiple bacterial taxa [14, 15]. In contrast, the viral component of this ecosystem—the gut virome—has received little attention. Most gut viruses are bacteriophages, particularly members of the class *Caudoviricetes*, which dominate in healthy individuals [16, 17]. These phages shape bacterial communities through lysis, horizontal gene transfer, and lysogenic infection [18–22]. For instance, *Caudoviricetes* phages can influence Firmicutes bacteria (e.g., *Lactococcus*, *Streptococcus*), affecting folate and one-carbon metabolism that connect microbial activity to host physiology [23]. Furthermore, altered virome diversity has been reported in diabetes, inflammatory bowel disease, and autoimmune disorders [24–26], suggesting that viral changes may contribute to disease through interactions with bacteria and metabolites. Phages can also influence host immunity directly [27, 28]. Nevertheless, the features of the gut virome and its interactions with bacterial communities and host metabolism in IPAH remain unclear.

In this study, we integrated metagenomic sequencing and metabolomic profiling to systematically compare gut virome composition between patients with IPAH and healthy controls (HCs). By dissecting virus–bacteria–metabolite interaction patterns, we aimed to elucidate the potential contribution of the gut virome to IPAH pathogenesis and uncover new mechanistic insights into its pathogenesis.

## Methods

### Study cohort

Between November 2023 and August 2024, we prospectively recruited 28 IPAH patients and 30 age- and sex-matched HCs from Beijing Chaoyang Hospital, Capital Medical University. The diagnosis of IPAH followed the most recent international guidelines. Participants were excluded if they were younger than 18 or older than 70 years, had a positive acute vasodilator response during right heart catheterization, or presented with major comorbidities such as coronary artery disease, pneumonia, diabetes, or chronic kidney disease. Individuals with a family history of autoimmune disorders, cancer, or other hereditary diseases were also excluded, as were those who had taken antibiotics within three months before enrollment. HCs were recruited from the hospital’s health examination center and confirmed to be free of cardiopulmonary or systemic diseases through clinical assessments. The study was approved by the Ethics Committee of Beijing Chaoyang Hospital (2023-ke-726), and the protocol complied with the Declaration of Helsinki. Written informed consent was obtained from all participants.

### Metagenomic sequencing

Total DNA was extracted from fecal samples using the cetyltrimethylammonium bromide method. DNA purity, concentration, and quality were assessed using NanoDrop spectrophotometry, Qubit 2.0 fluorometry, and agarose gel electrophoresis, respectively. Libraries were prepared and sequenced on the Illumina NovaSeq 6000 platform (paired end 2 × 150 bp) by Oriental Yeekang Medicine Technology Co., Ltd. Samples meeting quality control thresholds were used for downstream metagenomic analyses.

### Gut virome analysis

Raw paired-end reads were trimmed and quality-filtered using Trimmomatic v0.36 [29]. Host-derived sequences were removed by aligning reads to the human reference genome with the Burrows-Wheeler Aligner v0.7.12-r1039 [30], yielding high-quality clean reads.De novo assembly was performed using MegaHit v1.1.1–2-g02102e1 [31] with a minimum contig length of 500 bp. Contigs ≥ 2000 bp were subjected to viral identification using DeepVirFinder v1.1 [32], VirSorter2 v2.0 [33], VIBRANT v1.2.1 [34], and the Integrated Microbial Genomes/Virus (IMG/VR) database. Sequences supported by at least two tools were retained. Viral genome completeness was assessed using CheckV [35]. Contigs classified as “Not determined” were excluded from subsequent analyses.Candidate viral sequences were clustered into vOTUs using PhageAnnotator, with sequences sharing > 95% nucleotide identity across ≥ 85% of the shorter sequence length considered a single vOTU [36]. The longer sequence was designated as the representative sequence. To minimize bacterial contamination, single-copy bacterial marker genes were screened using Benchmarking Universal Single-Copy Orthologs (BUSCO) analysis [37]. BUSCO has been widely applied in virome studies as a complementary quality-control step to detect and remove contigs harboring bacterial conserved genes, thereby improving the specificity of viral genome reconstruction [37–39]. Only sequences with complete absence of bacterial single-copy orthologs were retained. Viral taxonomic annotation and host prediction were performed using VPF-Class [40].

### Gut bacteriome analysis

Taxonomic profiling of bacterial communities was conducted using MetaPhlAn4 v4.0.6 (release date: March 1, 2023), generating relative abundances across phylum to species levels under default parameters.

### Microbial diversity and differential abundance analysis

Permutational multivariate analysis of variance (PERMANOVA) was performed using the adonis2 function in the vegan R package (999 permutations) to assess the effects of host factors (IPAH status, age, sex, and body mass index [BMI]) on gut virome composition. The analysis was based on Bray–Curtis dissimilarity calculated from relative abundance profiles. A marginal (type III) sum-of-squares model was applied using the by = "margin" option to ensure that effect size estimation was independent of variable ordering. The model was specified as: Bray–Curtis distance ~ IPAH status + age + sex + BMI. Effect sizes were reported as R^2^. Alpha-diversity metrics, including Shannon, Simpson, Chao1, ACE, and Pielou’s evenness indices, were calculated using the phyloseq R package. Differences in alpha diversity between groups were assessed using the Mann–Whitney U test. Beta diversity was evaluated using Bray–Curtis dissimilarity derived from relative abundance profiles.

Community structure was visualized using principal coordinates analysis (PCoA) and non-metric multidimensional scaling (NMDS). Redundancy analysis (RDA) was performed to further assess the contribution of host factors to virome variation. To reduce sparsity in the feature table, taxa with prevalence < 20% across samples were excluded prior to downstream analyses. Differential abundance of viral features was assessed at both the vOTU and taxonomic levels using Analysis of Composition of Microbiomes with Bias Correction (ANCOM-BC), adjusting for age, sex, and BMI. Differential bacterial features between IPAH patients and healthy controls were similarly identified using ANCOM-BC, with taxonomic classification performed at the species level.

### Metabolomics analysis

Blood samples were collected in EDTA tubes, centrifuged, and plasma was stored at − 80 °C. Metabolites were profiled using ultra-high-performance liquid chromatography coupled with quadrupole time-of-flight mass spectrometry under hydrophilic interaction liquid chromatography (HILIC) conditions. Data processing, including peak detection and alignment, was performed using the XCMS R package, and metabolite annotation was conducted with CAMERA and an in-house database. Group discrimination was assessed by orthogonal partial least squares discriminant analysis (OPLS-DA), with variable importance in projection (VIP) > 1 and *p* values < 0.05 defining significant metabolites. Kyoto Encyclopedia of Genes and Genomes (KEGG) pathway analysis was used to perform functional enrichment.

### Statistical analysis and visualization

All statistical analyses were performed using R version 4.4.1 and GraphPad Prism version 10.1.2. Group comparisons of continuous clinical variables were performed using two-tailed Student’s t tests or Mann–Whitney U tests, as appropriate. Spearman’s rank correlation was used to assess associations among virus–virus, virus–bacterium, and bacteria–metabolite relationships. Network analyses and Sankey plots were constructed based on significant correlations. Machine learning analysis was performed using random forest models with fivefold cross-validation to evaluate the discriminatory performance of virome features. Model performance was assessed using receiver operating characteristic (ROC) curves and area under the curve (AUC) values. Statistical significance was defined as a two-sided *P* value < 0.05 unless otherwise specified.

## Results

### Overview of the gut virome in IPAH patients and HCs

Baseline characteristics of the study cohort are provided in Table 1. Age and sex were comparable between IPAH patients and healthy controls, minimizing potential confounding in downstream microbiome analyses. Metagenomic sequencing of fecal samples from 28 IPAH patients and 30 HCs generated 604.2 Gbp of high-quality data (10.4 ± 1.5 Gbp per sample; Table S1). De novo assembly yielded 5,750,160 contigs ≥ 500 bp (mean, 2521.3 bp; range, 500–1,043,951 bp). From 225,553 contigs ≥ 2000 bp, viral identification and CheckV assessment retained 79,465 viral sequences. Clustering yielded 34,947 vOTUs, and removal of bacterial contamination using BUSCO left 31,743 vOTUs for annotation. Of these, 42.1% (13,379/31,743) were taxonomically classified (Figure S1A). At the phylum level, *Uroviricota* accounted for > 99% of sequences in both groups (Figure S1B). At the class level, *Caudoviricetes* dominated (> 99%; Figure S1C), with *Caudoviricetes*_unclassified and *Crassvirales*_unclassified predominating at the family level (Figure S1D).

**Table 1:** Baseline demographic and clinical characteristics of healthy controls and IPAH patients

	CON (*n* = 30)	IPAH (*n* = 28)	*P-value*
Age, years	34.9 (26.0–49.0)	38.7 (29.3–44.0)	0.07
Male sex, n (%)	16(53.3)	13(46.4)	0.79
Cardiac Index (L/min/㎡)	NA	2.6 ± 0.5	NA
Pulmonary Vascular Resistance (WU)	NA	752.6 ± 226.5	NA
Pulmonary Artery Wedge Pressure (mmHg)	NA	10.6 ± 4.8	NA
Mean Pulmonary Artery Pressure (mmHg)	NA	47.7 ± 9.4	NA
Right Ventricular Systolic Pressure (mmHg)	NA	73.1 ± 15.0	NA
Mean Right Atrial Pressure (mmHg)	NA	10.1 ± 4.2	NA
Cardiac Output (L/min)	NA	4.2 ± 1.0	NA
NT-proBNP (pg/mL)	NA	2574.7 ± 3380.0	NA
6MWD (m)	NA	360.2 ± 96.2	NA
TAPSE (mm)	NA	8.1 ± 7.0	NA

Data are presented as mean ± SD, median (IQR), or n (%). *P*-values compare IPAH vs HC

*Abbreviations*: *IPAH* Idiopathic pulmonary arterial hypertension, *HC* Healthy control, *BMI* Body mass index, *6MWD* 6-min walk distance, *TAPSE* Tricuspid annular plane systolic excursion, *NA* Not applicable

### IPAH diagnosis as a key determinant of gut DNA virome composition

To delineate the characteristics of the gut virome in IPAH, we incorporated IPAH diagnosis, sex, age, and BMI as covariates to identify factors shaping virome composition. Analyses were performed at both the viral family and vOTU levels.

**Fig. 1:**
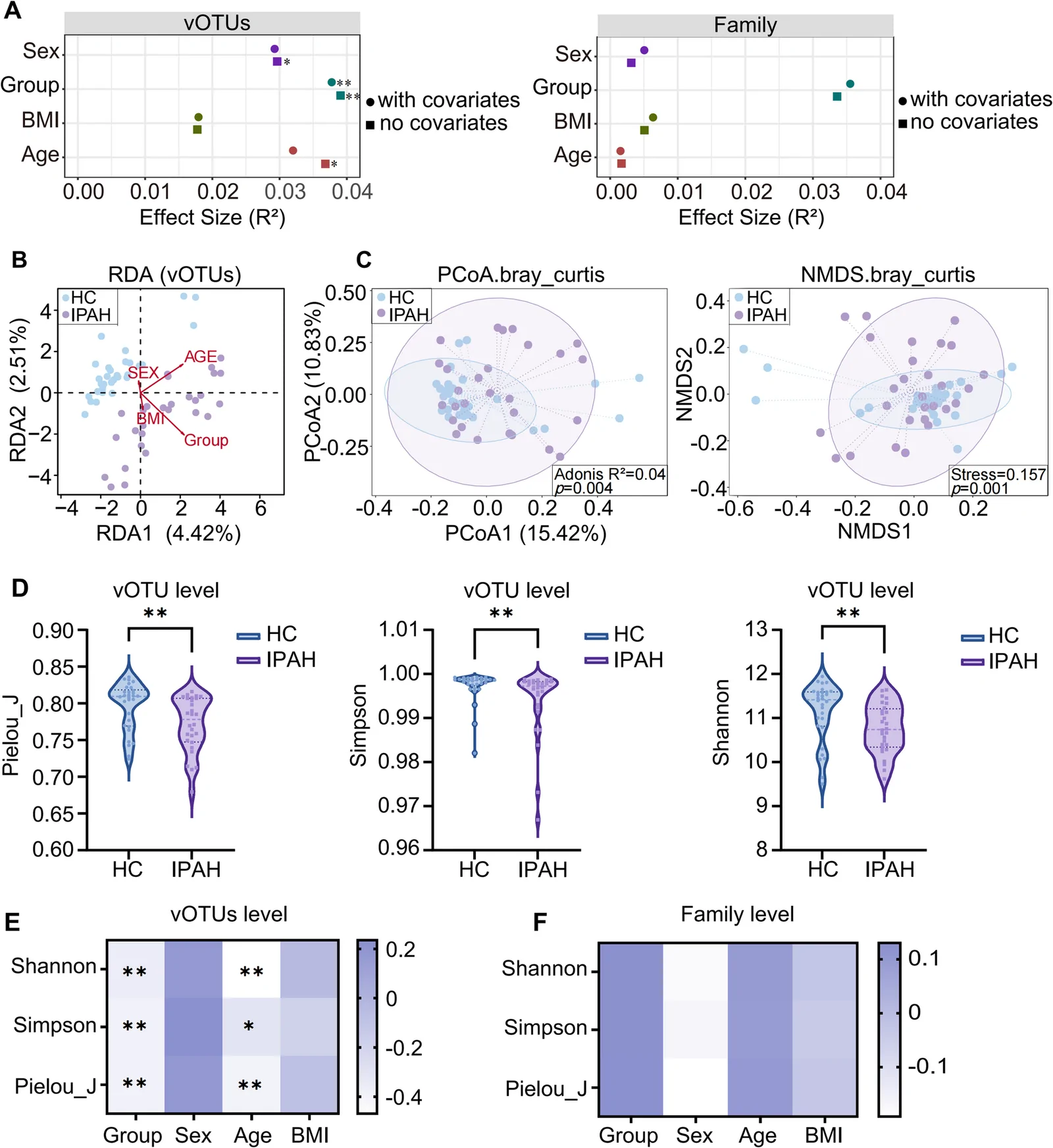
Analyses of gut virome variance and diversity in IPAH. **A** Variance in fecal virome composition explained by IPAH status and host factors (age, sex, BMI) at vOTU and family levels; effect sizes shown with and without covariates (circles and squares); *p < 0.05, **p < 0.01. **B** RDA showing directional effects of host factors on virome composition (arrows indicate direction and magnitude). **C** Beta-diversity (PCoA, NMDS) based on Bray–Curtisdistance; axes 1 and 2 reflect group-level variation. **D** Alpha-diversity metrics (Pielou evenness, Simpson, Shannon); *p* values from Mann–Whitney tests. **E**, **F** Heatmaps of Spearman’s correlations between host factors and alpha-diversity at the vOTU (**E**) and family levels (**F**); *p<0.05, **p<0.01

Multivariate analysis revealed that IPAH explained a significant proportion of gut viral community variation at the vOTU level (PERMANOVA, R^2^ = 3.91%, *P* = 0.005; Fig. 1A), emerging as the strongest covariate with the largest effect size. Associations of IPAH diagnosis, age, sex, and BMI with virome composition were more pronounced at the vOTU level than at the viral family level (e.g., IPAH diagnosis: vOTU R^2^ = 3.91%, *P* = 0.005 vs. family R^2^ = 3.55%, *P* = 0.135; Fig. 1A), highlighting differences between taxonomic family- and vOTU-based approaches in capturing host–virome associations. Community-level comparisons further demonstrated significant differences in gut viral composition between IPAH patients and HCs (Bray–Curtis dissimilarities; Fig. 1B). Principal coordinates analysis (PCoA) revealed a clear separation of IPAH and HC samples, consistent with results from non-metric multidimensional scaling (NMDS; Fig. 1C). Beyond IPAH diagnosis, age and sex were also correlated with virome variation across all subjects. However, no significant differences in age or sex were observed between IPAH patients and HCs within our cohort (Mann–Whitney test).

Viral alpha-diversity was assessed at both the vOTU and viral family levels. At the family level, Shannon, Simpson, and Pielou’s evenness indices did not differ significantly between IPAH patients and HCs, nor were they associated with host factors (Fig. 1F). In contrast, IPAH patients exhibited significantly reduced Shannon, Simpson, and Pielou’s evenness indices at the vOTU level (Fig. 1D), all of which were significantly correlated with IPAH diagnosis and age (Spearman’s rank correlation; Fig. 1E).

Collectively, these findings demonstrated that, compared with HCs, IPAH patients exhibit marked alterations in gut viral community composition and structure, accompanied by significantly reduced viral diversity.

### Altered gut virus–virus interactions and increased abundance of *Uroviricota* in IPAH patients

**Fig. 2:**
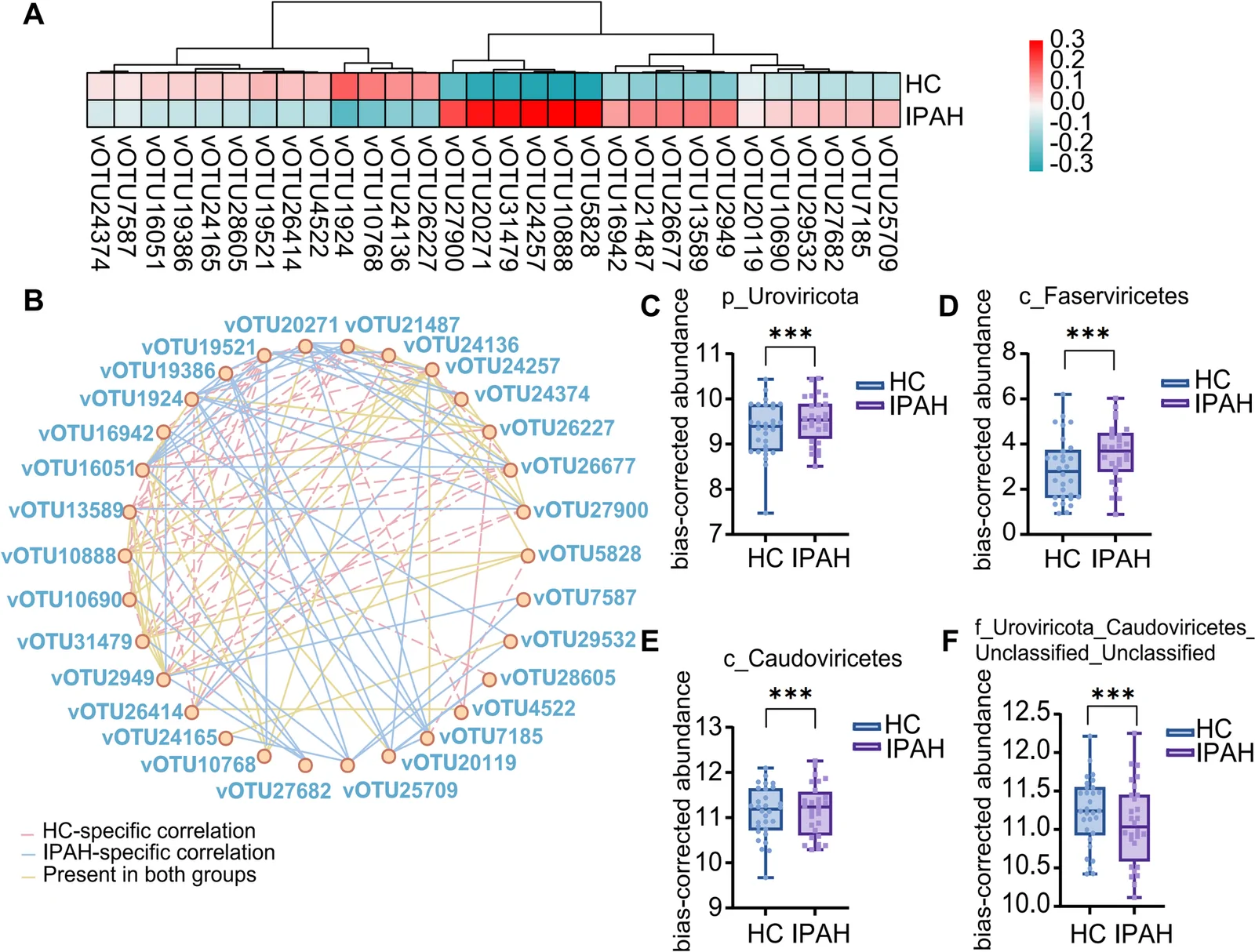
Altered gut viral ecology in IPAH. **A** Heatmap depicting the relative abundance and distribution of the 30 most differentially abundant vOTUs across groups, identified via ANCOM-BC. **B** Gut viral community network showing significantly altered correlations between IPAH patients and HCs; *p* < 0.05. **C**–**F** Differentially abundantviruses between the two groups at various taxonomic levels, identified by ANCOM-BC after adjusting for age, sex, and BMI

ANCOM-BC identified 499 differentially abundant vOTUs after adjusting for age, sex, and BMI. Heatmap analysis of the top 30 vOTUs revealed clear group-level clustering (Fig. 2A). Network analysis of these vOTUs showed altered virus–virus interactions, including numerous IPAH-specific associations (Fig. 2B). Taxonomic profiling further revealed increased abundance of *Uroviricota* at the phylum level and enrichment of *Faserviricetes* and *Caudoviricetes* at the class level, with *Uroviricota*_*Caudoviricetes* (unclassified, unclassified) enriched at the family level in IPAH patients (Fig. 2C–F).

### Significant associations between gut viral features and IPAH clinical indicators

Correlation analyses linked differential vOTUs with clinical parameters. Of 499 differentially abundant vOTUs, 42 were significantly correlated with at least one IPAH clinical indicator (*P* ≤ 0.05; Figure S2A). *Uroviricota* abundance was negatively correlated with mean pulmonary arterial pressure (mPAP; Figure S2B), suggesting a potential protective role. At the family level, *Uroviricota*_*Caudoviricetes* (unclassified, unclassified) was positively correlated with tricuspid annular plane systolic excursion (TAPSE), an index of right ventricular function. These findings indicated that differentially abundant viruses across multiple taxonomic levels are linked to IPAH clinical metrics and may serve as biomarkers or therapeutic targets.

### Altered gut virus–bacterium networks in IPAH

**Fig. 3:**
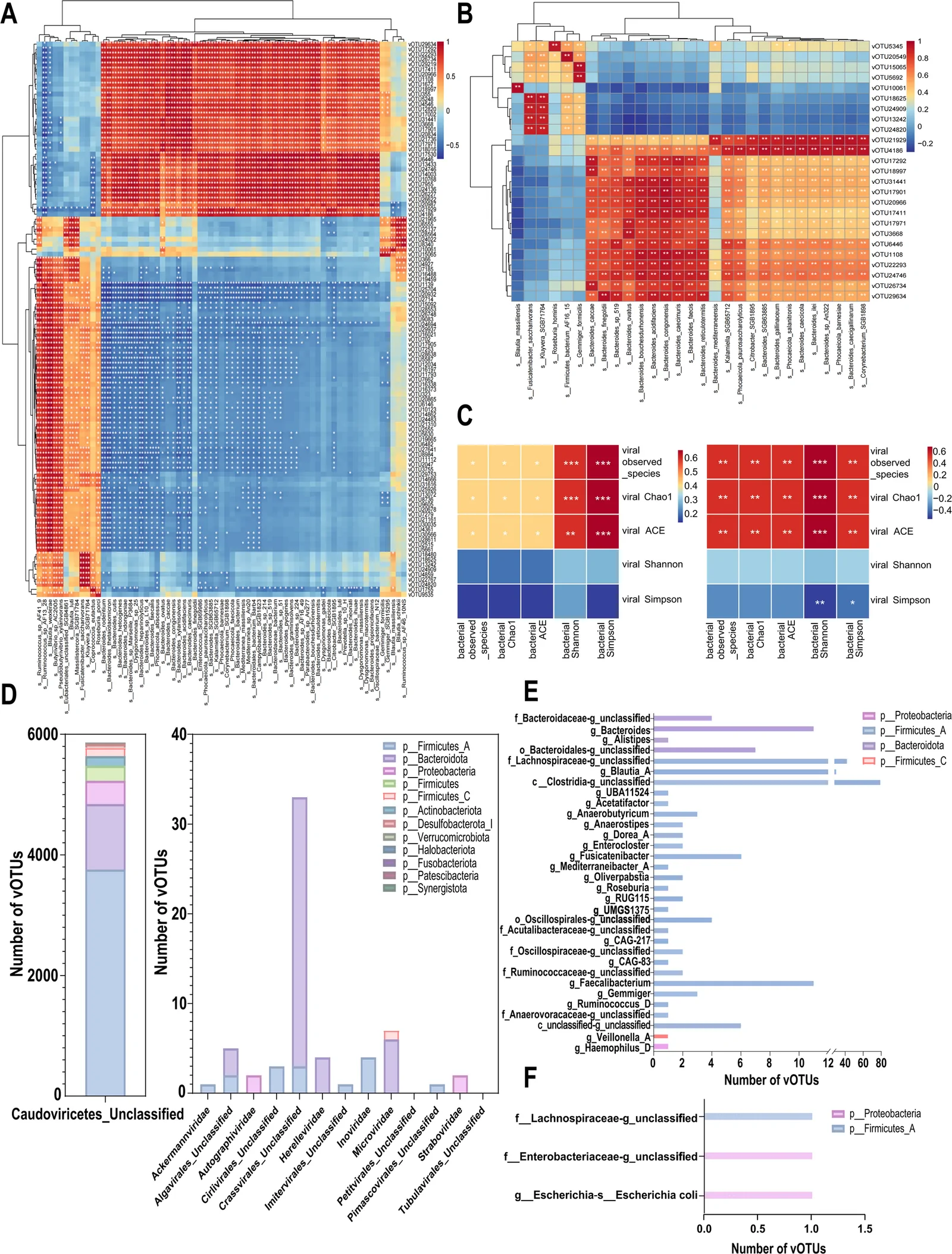
Altered gut virus–bacteria networks in IPAH. **A**, **B** Strong correlations (|r|>0.9) between differential vOTUs and bacteria in IPAH patients (**A**) and HCs (**B**). **C** Altered á-diversity correlations: viral observed_species, Chao1, and ACE positively correlated with bacterial indices (*p*<0.001), while Simpson indices showed negative correlations (*p*<0.05). (D) Host prediction assigned 44.3% of vOTUs, primarily to Firmicutes, Bacteroidota, Proteobacteria, and Actinobacteriota. **E**, **F** Enriched vOTUs in IPAH patients were predominantly linked to Firmicutes, while two-thirds of depleted vOTUs were linked to Proteobacteria; *p<0.05, **p<0.01

To elucidate the potential interplay between gut viruses and bacteria in IPAH, we conducted differential and correlation analyses. ANCOM-BC identified 666 bacterial species that were differentially abundant between IPAH patients and HCs. At the vOTU level, Spearman’s correlation analysis revealed 44,894 strong virus–bacterium associations (*r* > 0.5, *P* < 0.05) in IPAH, markedly exceeding the 17,920 associations observed in HCs. Focusing on associations between differential viruses (phylum/class/family levels) and differential bacteria, the IPAH group also exhibited stronger connectivity (144 vs. 119 in HCs). Heatmap visualization of correlations with |*r*|> 0.9 highlighted pronounced shifts in the association patterns between differential vOTUs and bacteria in IPAH patients compared with those in HCs (Fig. 3A and 3B). Notably, in HCs, *Uroviricota* showed significant correlations with only nine differential bacterial species, whereas in IPAH this increased to 60 strong associations. Similarly, the viral classes *Faserviricetes* and *Caudoviricetes* exhibited eight and five strong associations in HCs, respectively, increasing to 31 and 22 in IPAH patients, with six associations shared between groups.

Analysis of virus–bacterium interactions further revealed significantly altered alpha-diversity correlation patterns in IPAH patients. The observed species, Chao1, and ACE indices for viruses remained strongly positively correlated with their bacterial counterparts (*P* < 0.001), with greater strength than in HCs. In contrast, viral and bacterial Simpson indices were significantly negatively correlated (*P* < 0.05) (Fig. 3C).

Host prediction analysis showed that 44.3% (5927/13,379) of vOTUs could be assigned to one or more bacterial hosts, predominantly from the phyla Firmicutes, Bacteroidota, Proteobacteria, and Actinobacteriota (Fig. 3D), consistent with previous reports of broad viral host distributions in the gut [41]. Among vOTUs with predicted hosts, the majority of significantly enriched vOTUs in IPAH patients were assigned to Firmicutes, with only one vOTU assigned to Proteobacteria (Fig. 3E). Conversely, among the few significantly reduced vOTUs in IPAH patients with host assignments, two of three were linked to Proteobacteria (Fig. 3F).

**Fig. 4:**
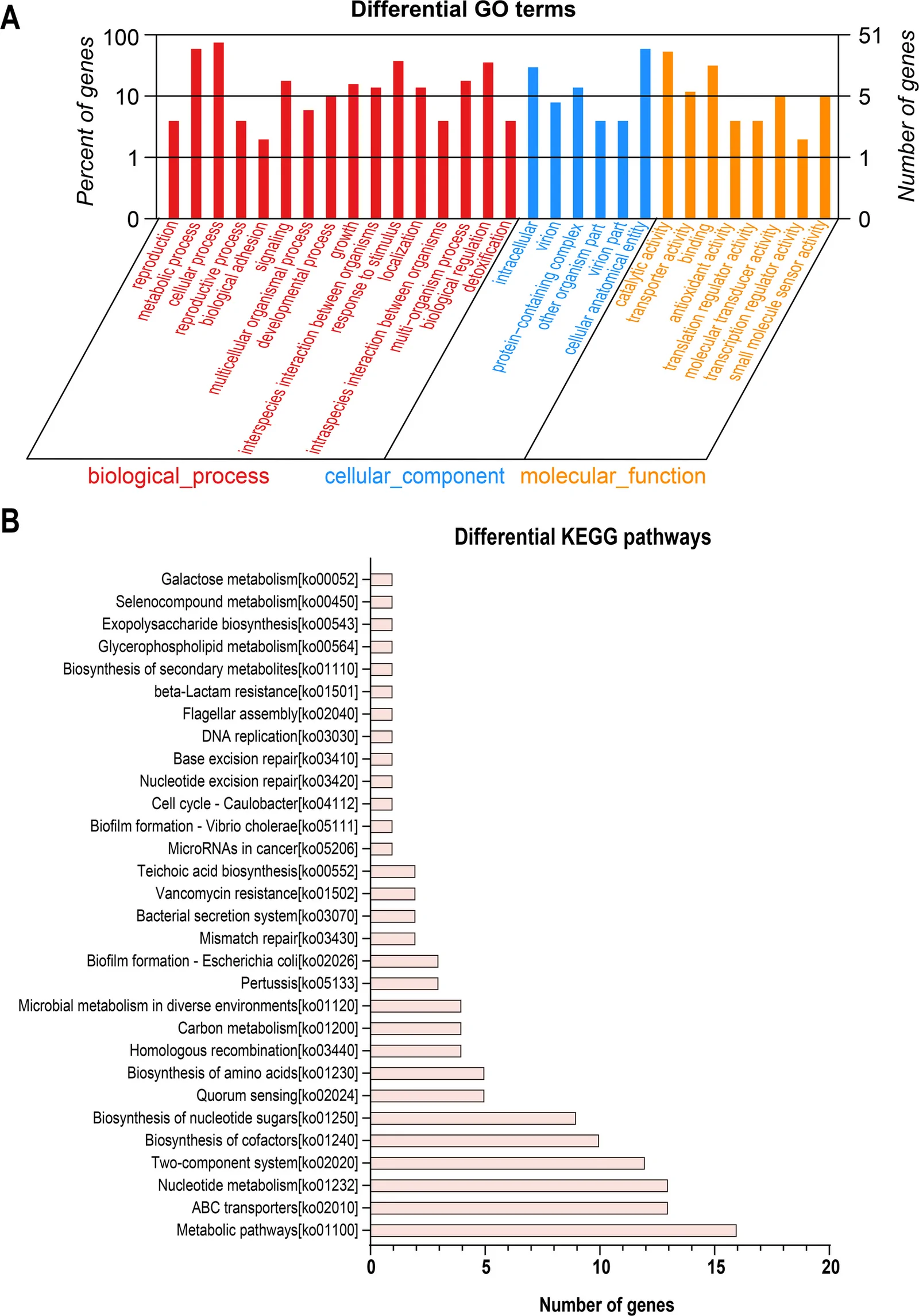
Functional alterations of the gut virome in IPAH. **A** Thirty significantly altered GO terms (biological process, cellular component, and molecular function), dominated by metabolic and cellular processes, catalytic activity, and binding. **B** Thirty significantly altered KEGG pathways, including those involved in metabolism, ABC transporters, nucleotide metabolism, two-component systems, and cofactor biosynthesis, suggesting roles in disease progression

A considerable proportion of enriched vOTUs in IPAH patients were predicted to target the genus *Blautia*. In HCs, s__*Blautia obeum* was significantly positively correlated with *Uroviricota*_*Caudoviricetes* (unclassified, unclassified) viruses (ρ = 0.604, *P* < 0.001). In contrast, s__*B. obeum* abundance was markedly reduced in IPAH patients, while the corresponding viral taxa were significantly increased, resulting in a weakened and nonsignificant association. This disruption suggested a breakdown in the coordinated relationship between viruses and *Blautia* in the gut. Representing a potential host for *Uroviricota*_*Caudoviricetes* viruses, the genus *Blautia* also accounted for a substantial fraction of host assignments among enriched vOTUs in IPAH patients. As a beneficial commensal, *Blautia* contributes to gut homeostasis and regulation of inflammation via short-chain fatty acid production [42–45]. In disease states, expansion of *Blautia*-targeting viruses may reduce *Blautia* abundance (including *B. obeum*) or impair its functions through lysis, inhibition of growth, or disruption of metabolic activity, thereby destabilizing gut ecology and contributing to disease progression.

A similar pattern was observed for the viral family *Imitervirales* (unclassified), which showed a positive correlation with s__*Anaerostipes hadrus* in both groups, with a stronger association in IPAH patients. Based on IMG/VR annotations from metagenomic sequencing, *Anaerostipes* was identified as the predicted bacterial host of this virus.

Collectively, these findings suggested that viruses may participate in gut ecological regulation by targeting specific bacterial hosts. Virus–bacterium interactions may jointly influence the development and progression of IPAH.

### Altered viral functions in IPAH patients, with significant upregulation of viral *lgoD*

To investigate functional changes in the gut virome, viral gene prediction was performed using MetaProdigal (http://prodigal.ornl.gov/), followed by annotation of predicted protein sequences against the KEGG and Gene Ontology (GO) databases. Viral sequence and gene abundances were quantified using the Burrows-Wheeler Aligner, enabling comparative analyses of viral gene functions between IPAH patients and HCs.

Alterations in viral functional profiles were observed in IPAH patients, with 30 GO terms (Fig. 4A) and 30 KEGG pathways (Fig. 4B) reaching statistical significance. Enriched GO terms spanned all three major categories: biological process, cellular component, and molecular function. Within biological processes, genes involved in metabolic processes and cellular processes were particularly prominent. At the molecular function level, catalytic and binding activities were key enriched features, while the cellular component category displayed specific functional distributions. KEGG pathway analysis revealed differential expression of metabolic pathways, ABC transporters, nucleotide metabolism, two-component systems, and biosynthesis of cofactors, suggesting potential contributions to disease progression.

Notably, metagenomic analysis revealed significant upregulation of the viral-derived L-galactonate-5-dehydrogenase (*lgoD*) gene in IPAH patients compared with that in HCs (*P* < 0.05). Based on KEGG pathway annotation, *lgoD* encodes a key enzyme in the classical ascorbate biosynthesis pathway via the L-galactose route, catalyzing the conversion of L-galactonic acid to an ascorbate precursor. Despite the elevated expression of *lgoD* in IPAH patients, serum ascorbate levels were markedly reduced. Previous studies have demonstrated that vitamin C deficiency can induce PAH, while supplementation with vitamin C reverses PAH induced by ascorbate deficiency [46].

### Distinct lytic and temperate phage distributions in the gut viromes of IPAH patients and HCs

Phage lifestyle prediction using VIBRANT classified 10,633/31,743 vOTUs (33.50%) as lytic or temperate, while 21,110 remained unassigned due to missing marker genes or reference sequences (Figure S3). Among the 499 differential vOTUs, 48 (9.62%) were classifiable: 25 temperate phages (52.08%), all upregulated in IPAH, including five assigned to *Caudoviricetes* (Firmicutes hosts); and 23 lytic phages (47.92%), of which 21 were upregulated and two (vOTU9018, vOTU6641) downregulated. Host prediction linked downregulated vOTU9018 to a Caudoviricetes phage infecting Clostridia hosts, while eight of the upregulated lytic vOTUs were linked to *Caudoviricetes*, infecting members of Firmicutes and Bacteroidota.

### Associations between gut viruses, bacteria, and serum metabolite levels in IPAH

**Fig. 5:**
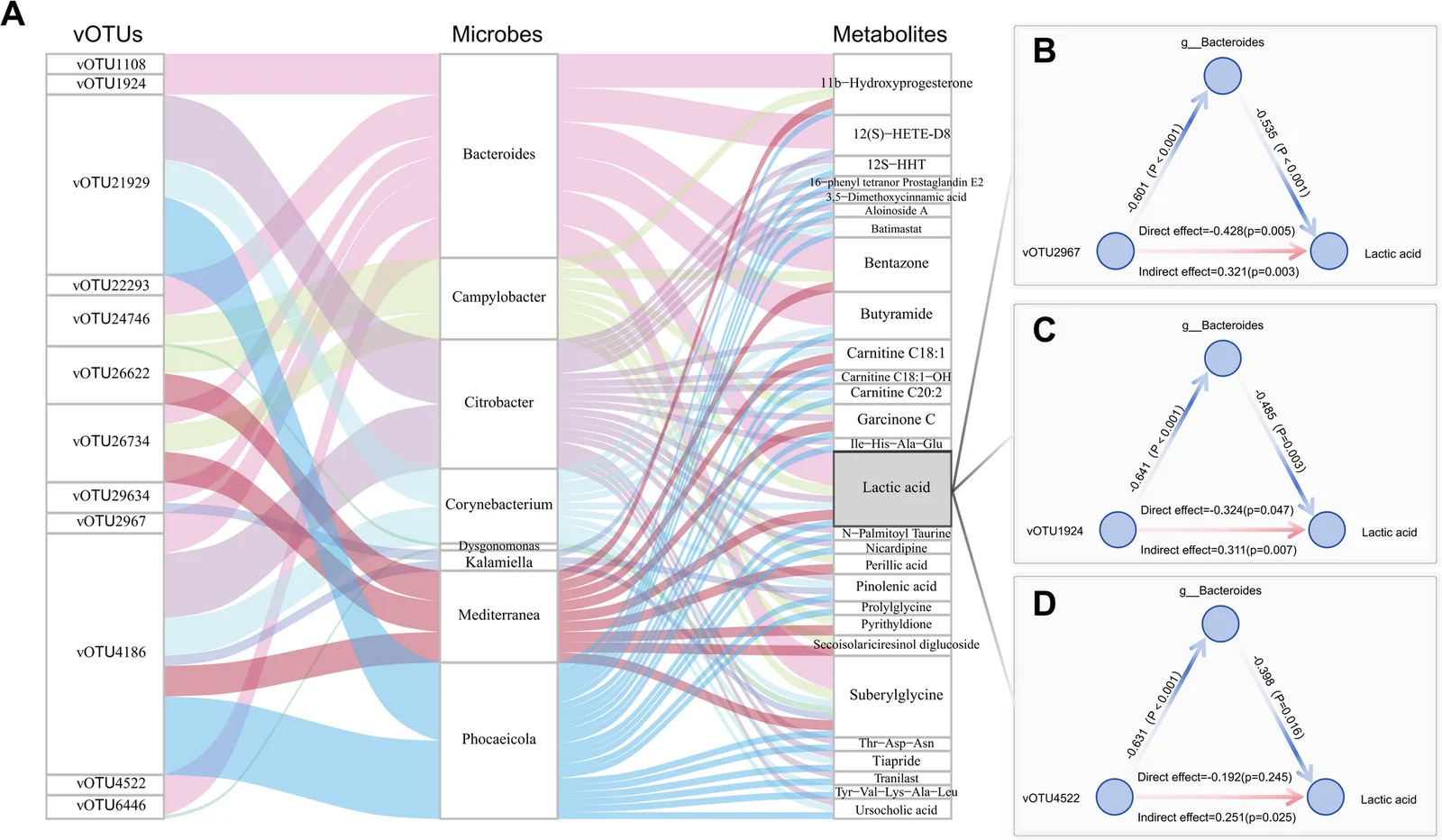
Virus–bacterium–metabolite mediation in IPAH. **A** Sankey diagram showing significant mediating links among gut vOTUs, bacterial taxa, and serum metabolites; curves reflect regulatory effects. **B**–**D** Representativemediation pathways with labeled beta coefficients and significance; red arrows indicate positive regulation, blue arrows negative regulation

Metabolites derived from the gut microbiota are recognized as key mediators through which bacteria exert profound effects on host organs and physiology [47]. In patients with PAH, gut dysbiosis has been associated with a proinflammatory metabolic profile, including reduced short-chain fatty acids and elevated lactate and bile acid derivatives, which may contribute to endothelial dysfunction and vascular remodeling [48]. Untargeted serum metabolomics identified 1,374 differentially abundant metabolites between IPAH patients and HCs (VIP > 1, *P* < 0.05; Figure S4). A virus–bacterium–metabolite Sankey diagram illustrated structured interaction patterns (Fig. 5A). Mediation analysis identified 15 significant virus–bacterium–metabolite pathways (Figs. 5B–D and S5), including lactic acid, suberylglycine, butyramide, and 12(S)-HETE-D8. Enriched vOTUs (vOTU2967, vOTU1924, vOTU4522) were predicted to negatively regulate *Bacteroides*, which in turn suppressed the lactic acid pathway, yielding significant indirect effects (*P* < 0.05). Collectively, these interactions contributed to a net accumulation of lactic acid in IPAH patients, consistent with previously reported elevated lactate phenotypes in PAH, where serum lactic acid levels are associated with disease severity [49].

Beyond lactate, other pathways linked vOTUs to suberylglycine, butyramide, and 12(S)-HETE-D8. For example, vOTU1924 was associated with reduced *Bacteroides* abundance, which in turn was linked to increased levels of 12(S)-HETE-D8 (indirect effect = –0.446, *P* < 0.001). At the network level, these interactions formed a complex regulatory framework, implicating gut viruses in microbial metabolic balance and disease processes.

### Gut virome-based prediction of IPAH status

**Fig. 6:**
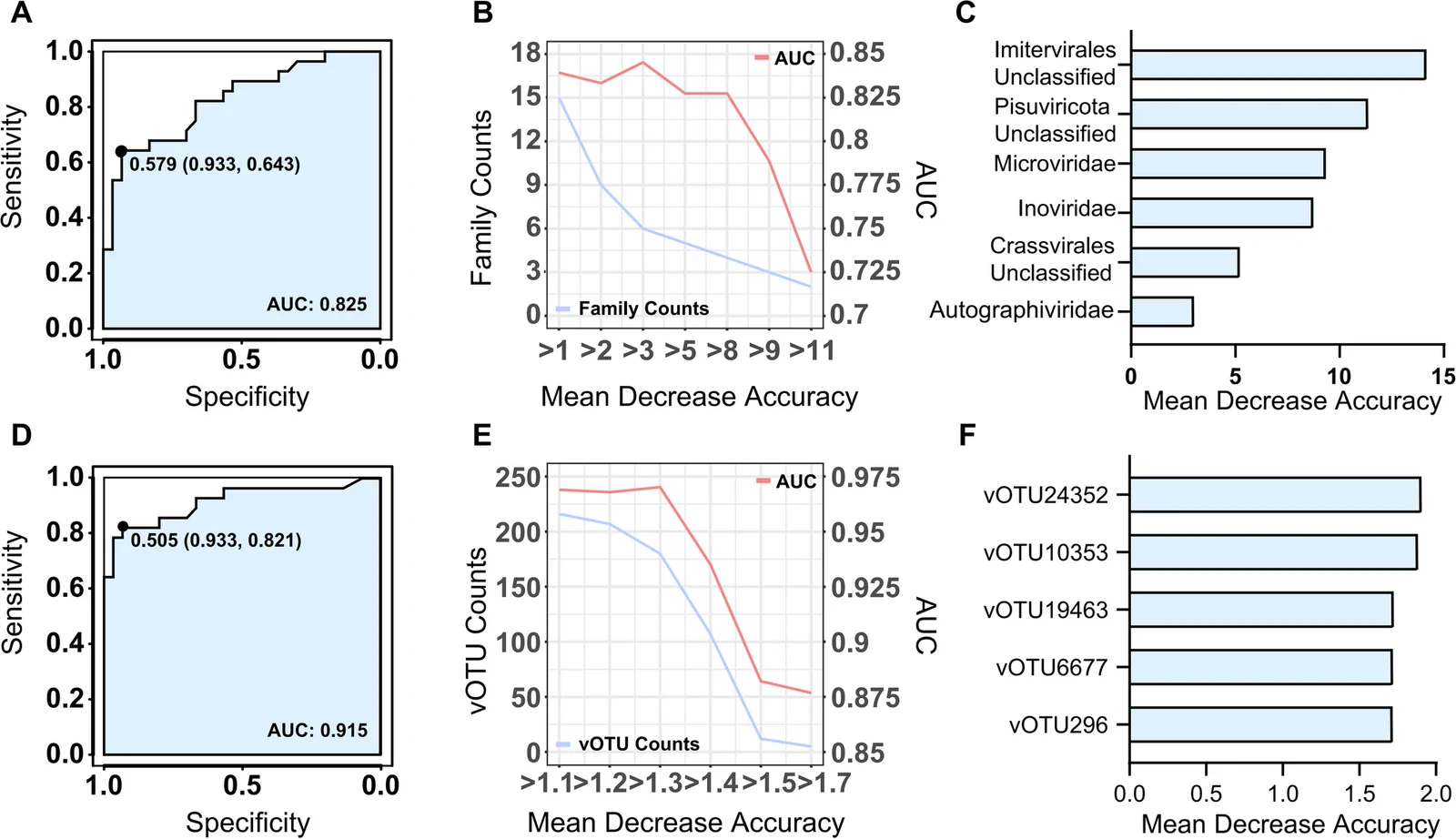
Gut virome-based classification of IPAH. **A**, **D** ROC curves for classification models using all viral families (**A**) and vOTUs (**D**). **B**, **E** AUC values across models with varying numbers of viral families (**B**) or vOTUs (**E**); red line indicates the number of families, blue line the AUC. **C**, **F** Key viral families (**C**) and vOTUs (**F**) ranked by the MDA from random forest analysis

To assess the discriminative capacity of the gut virome in IPAH, we constructed random forest models at both the viral family and vOTU levels. Using all viral families, the model reached an AUC of 0.825 (Fig. 6A). Refinement to the six most informative families raised the AUC to 0.845 (Fig. 6B), demonstrating strong discriminatory power (Fig. 6C). At the vOTU level, inclusion of all vOTUs yielded an AUC of 0.915 (Fig. 6D). Selection of 180 vOTUs with a mean decrease in accuracy (MDA) > 1.3 further increased the AUC to 0.970 (Fig. 6E). A stricter threshold (MDA > 1.7) reduced the panel to five vOTUs, resulting in an AUC of 0.830 (95% confidence interval: 0.723–0.936; Fig. 6F). These findings highlight the potential of the gut virome to discriminate IPAH patients from healthy controls, with vOTU-level features providing higher predictive resolution than family-level profiles. While promising, these analyses are exploratory and intended to generate hypotheses regarding virome-associated mechanisms rather than for clinical diagnosis.

## Discussion

In the present study, we provided the first systematic characterization of the gut virome in IPAH, demonstrating reduced diversity, significant dysregulation of viral taxa, and a marked reconfiguration of virus–bacteria interaction networks. Importantly, analyses at the level of vOTUs identified hundreds of differentially abundant viral signatures, several of which were strongly linked to bacterial taxa and metabolic alterations, suggesting a novel mechanism through which viruses may contribute to disease progression. Furthermore, virome-based machine learning models achieved robust discrimination between IPAH patients and controls.

Although direct evidence linking the gut virome to IPAH remains limited, accumulating evidence from other disease contexts indicates that bacteriophages can actively shape bacterial communities and metabolic outputs [50–52]. Phages regulate bacterial populations through lytic activity, lysogenic conversion, and horizontal gene transfer, thereby influencing microbial composition and function. Functional profiling further revealed altered distributions of lytic and temperate phages. Lytic phages rapidly lyse hosts, while temperate phages integrate into host genomes, where they can switch to lytic cycles under stress, thereby contributing to microbial stability and host adaptation [53, 54]. In IPAH patients, *Caudoviricetes* were enriched, encoding genes involved in nucleotide metabolism and transcription/translation, with integration potential that may promote chronic inflammation through persistent infection [55]. However, given the cross-sectional design, the directionality of these interactions cannot be definitively established, and bidirectional relationships between bacteriophages and their bacterial hosts remain possible.

A key observation in this study is the altered virus–bacterium relationships involving the commensal bacterium *Blautia*, a well-recognized short-chain fatty acid (SCFA)-producing taxon that contributes to intestinal homeostasis and regulation of inflammation [56–58]. Butyrate produced by these bacteria is known to modulate immune responses, enhance epithelial barrier integrity, and suppress pro-inflammatory signaling pathways [59–61]. While direct evidence in IPAH is currently lacking, the observed reduction in *Blautia* abundance alongside enrichment of certain vOTUs suggests a potential impact on SCFA-mediated homeostasis. For example, vOTU2564 was predicted to infect *Blautia*, indicating a plausible phage–host interaction. These observations support a hypothesis in which expansion of phages targeting SCFA-producing bacteria could influence microbial balance and immunometabolic regulation, but further experimental validation is required.

In parallel, our integrative analysis identified a virome-associated mechanism linked to lactate metabolism. Lactate is increasingly recognized as a key metabolic mediator in pulmonary arterial hypertension, contributing to pulmonary vascular remodeling, and elevated levels have been associated with disease severity and adverse hemodynamic profiles [62, 63].Mediation analysis suggested that specific vOTUs may indirectly influence lactate accumulation through suppression of bacterial taxa such as *Bacteroides*, supporting a virus–bacteria–metabolite axis in IPAH. In particular, vOTU4522 showed a negative association with *Bacteroides*, a genus involved in metabolic homeostasis and lactate turnover, while vOTU2967 and vOTU1924 exhibited similar patterns. While direct evidence in IPAH is currently lacking, studies in non-IPAH contexts provide mechanistic support for a virus–bacteria–metabolite axis. For instance, in a human gut model, the temperate Bacteroides phage BV01 was shown to substantially alter the transcriptional profile of its Bacteroides host [64], indicating that phage infection can modulate bacterial metabolic pathways, including those involved in lactate production. Moreover, phage-host interactions were found to be dependent on the metabolic state of the host bacteria [65], suggesting a bidirectional regulatory relationship between phages and bacterial metabolism. In germ-free and gnotobiotic mouse models, Bacteroides-targeting phages were further demonstrated to influence bacterial abundance and colonization dynamics [66, 67], indirectly affecting metabolic outputs such as short-chain fatty acids and lactate. These findings from non-IPAH studies support the plausibility of our observed associations between vOTU4522, vOTU2967, and vOTU1924 and Bacteroides-mediated lactate metabolism in IPAH, suggesting that phage-driven modulation of gut bacteria may contribute to systemic metabolic dysregulation.

Collectively, these findings suggest that gut virome alterations in IPAH may affect multiple bacterial functional groups and their associated metabolic outputs, including SCFA production and lactate metabolism, thereby linking phage-driven microbial remodeling to systemic metabolic dysregulation.

Functional profiling further revealed alterations in viral gene content, including significant upregulation of the *lgoD* gene involved in the L-galactose pathway of ascorbate biosynthesis. Vitamin C plays an important role in redox homeostasis and vascular function, and its deficiency has been associated with pulmonary hypertension in specific clinical contexts [68]. Despite increased abundance of viral *lgoD*, serum ascorbate levels were reduced in IPAH patients, suggesting a potential compensatory response to metabolic imbalance. However, the functional relevance of this observation remains to be elucidated.

In addition to mechanistic insights, we further evaluated the translational potential of the gut virome. Multivariate analysis showed a strong association between IPAH status and gut viral community structure, with vOTU-level features explaining a substantial proportion of virome variation. This likely reflects incomplete virome reference databases, which limit annotation of unclassified vOTUs. As minimal units of viral classification, vOTUs capture novel and low-abundance viral fragments that may mediate links between disease and virome composition, whereas family-level analyses overlook many functional signals, reducing explanatory power.

Building on these mechanistic insights, we evaluated whether specific virome signatures could discriminate IPAH patients from healthy controls. Machine learning analysis using random forest models indicated that virome-derived variables, especially vOTU-level features, could distinguish IPAH patients from healthy controls with high accuracy. A small panel of five vOTUs provided efficient classification. Because vOTUs can be measured from fecal samples and have been linked to various diseases [38, 69, 70], such signatures may have value for early detection or clinical monitoring of IPAH. Due to the relatively small sample size and high dimensionality of the dataset, the specific vOTUs selected as top predictors may vary depending on cross-validation folds and thresholds. All results reported here are based on fivefold cross-validation, which provides a more stable estimate of model performance. Nevertheless, the overall discriminatory performance remains robust, demonstrating the exploratory potential of vOTU-level features in IPAH.

This study has limitations. First, the relatively small sample size (28 IPAH patients vs. 30 HCs) may have constrained the detection of low-frequency viral markers; validation in larger, multicenter cohorts is required. Second, we employed bulk metagenomic sequencing rather than viral particle enrichment. While bulk metagenomics provides a comprehensive view of microbial communities—capturing viruses, bacterial hosts, and other microorganisms—the low proportion of viral sequences and resulting reduced sensitivity for viral detection may limit assembly completeness and diversity resolution [71]. Compared with viromics based on particle enrichment, bulk approaches often yield fragmented viral genomes. IPAH patients routinely receive medications (ERA, PDE5i, prostacyclins, diuretics, oxygen therapy), which may affect the microbiome. Because excluding treated patients is not feasible or clinically representative, this factor remains an unavoidable limitation and is acknowledged as such. Finally, the study establishes associations between virome alterations and IPAH, but does not address causality or underlying mechanisms. Experimental approaches, such as fecal virome transplantation in animal models, will be essential to validate the causal role of virome dysbiosis in IPAH pathogenesis.

## Conclusions

This study systematically characterizes the gut virome in IPAH, revealing reduced viral diversity, extensive alterations in vOTUs, enrichment of viral functional genes (e.g., *lgoD*), and disrupted interactions among viruses, bacteria, and metabolites, particularly involving a virus–*Bacteroides*–lactate pathway. These data extend microbiome research in pulmonary hypertension beyond bacteria, identifying viruses as key modulators of microbial and metabolic homeostasis, supporting the diagnostic potential of vOTU signatures, and highlighting the potential of virome-targeted strategies as novel therapeutic avenues.

## Supplementary Information

Supplementary Material 1.

## Data Availability

Metagenomics raw sequences generated in this study have been deposited in the NCBI Sequence Read Archive (SRA) under accession number PRJNA1344780 for human samples. Additional metagenomics raw sequences have been deposited in MetaboLights under accession number MTBLS14077. All other data supporting the findings of this study are available from the corresponding author upon reasonable request.

## Abbreviations

ANCOM-BC: Analysis of Composition of Microbiomes with Bias Correction
AUC: Area under the curve
BUSCO: Benchmarking Universal Single-Copy Orthologs
GO: Gene Ontology
HCs: Healthy controls
IPAH: Idiopathic pulmonary arterial hypertension
KEGG: Kyoto Encyclopedia of Genes and Genomes
LgoD: L-galactonate-5-dehydrogenase
MDA: Mean decrease in accuracy
mPAP: Mean pulmonary arterial pressure
NMDS: Non-metric multidimensional scaling
OPLS-DA: Orthogonal partial least squares discriminant analysis
PCoA: Principal coordinates analysis
PERMANOVA: Permutational multivariate analysis of variance
RDA: Redundancy analysis
ROC: Receiver operating characteristic
TAPSE: Tricuspid annular plane systolic excursion
VIP: Variable importance in projection
vOTU: Viral operational taxonomic unit
