# Supplementary material for: Alterations in the fecal virome and bacteriome–virome interplay in IPAH
Source: Respir Res. 2026 Jul 1;27:382. doi: 10.1186/s12931-026-03797-x (PMC13637390; doi:10.1186/s12931-026-03797-x)
Supplement: Supplementary file 1 [file 12931_2026_3797_MOESM1_ESM.pdf]

Table S1. Sequencing summary of IPAH patients and healthy controls in this study.

SampleID	Number of reads	Gbp
HC-27	69878236	10.44293552
HC-18	65490854	9.783333292
HC-19	70511636	10.53370663
HC-29	70212830	10.49059406
HC-31	70107170	10.47809806
HC-1	70566152	10.54541786
HC-4	68808428	10.28263785
HC-11	69781934	10.42637614
HC-16	70120132	10.47718315
HC-20	68469188	10.23246228
HC-15	71327334	10.65762227
HC-8	65745488	9.820981493
HC-22	65744734	9.822622546
HC-25	70774348	10.57815196
HC-28	70290346	10.50272891
HC-30	68382802	10.21595634
HC-6	69106454	10.32550824
HC-13	69319562	10.35724023
HC-7	69433032	10.37638617
HC-9	66520096	9.938117328
HC-3	69656186	10.40929051
HC-35	70722792	10.56809911
HC-39	69672064	10.41471787
HC-40	68446450	10.22601588
HC-38	69990776	10.45967656
HC-33	67847538	10.1410915
HC-36	70419708	10.52402766
HC-37	70459910	10.5303313
HC-32	70545904	10.54430692
HC-34	70585854	10.54873726
IPAH-1	70137332	10.47709255
IPAH-2	70534404	10.5364806
IPAH-3	69596802	10.39732743
IPAH-4	47198494	7.055507235
IPAH-5	69916816	10.4487899
IPAH-6	66007784	9.860442453
IPAH-7	70881538	10.5893377
IPAH-8	69013806	10.30416732
IPAH-9	70397742	10.51779058
IPAH-10	68545220	10.24308228
IPAH-11	70332922	10.51093822
IPAH-12	70492236	10.53315771
IPAH-13	64178130	9.585068641
IPAH-14	125639518	18.75875681
IPAH-17	65877032	9.843758968

IPAH-20	68250504	10.1964709
IPAH-21	64004284	9.56426796
IPAH-22	71254612	10.64393451
IPAH-23	57298816	8.558359055
IPAH-24	68201072	10.19066913
IPAH-25	76819738	11.48097872
IPAH-26	75328118	11.25638559
IPAH-28	77140504	11.52912959
IPAH-29	29288780	4.376093423
IPAH-30	84298024	12.58464061
IPAH-31	68893438	10.29054363

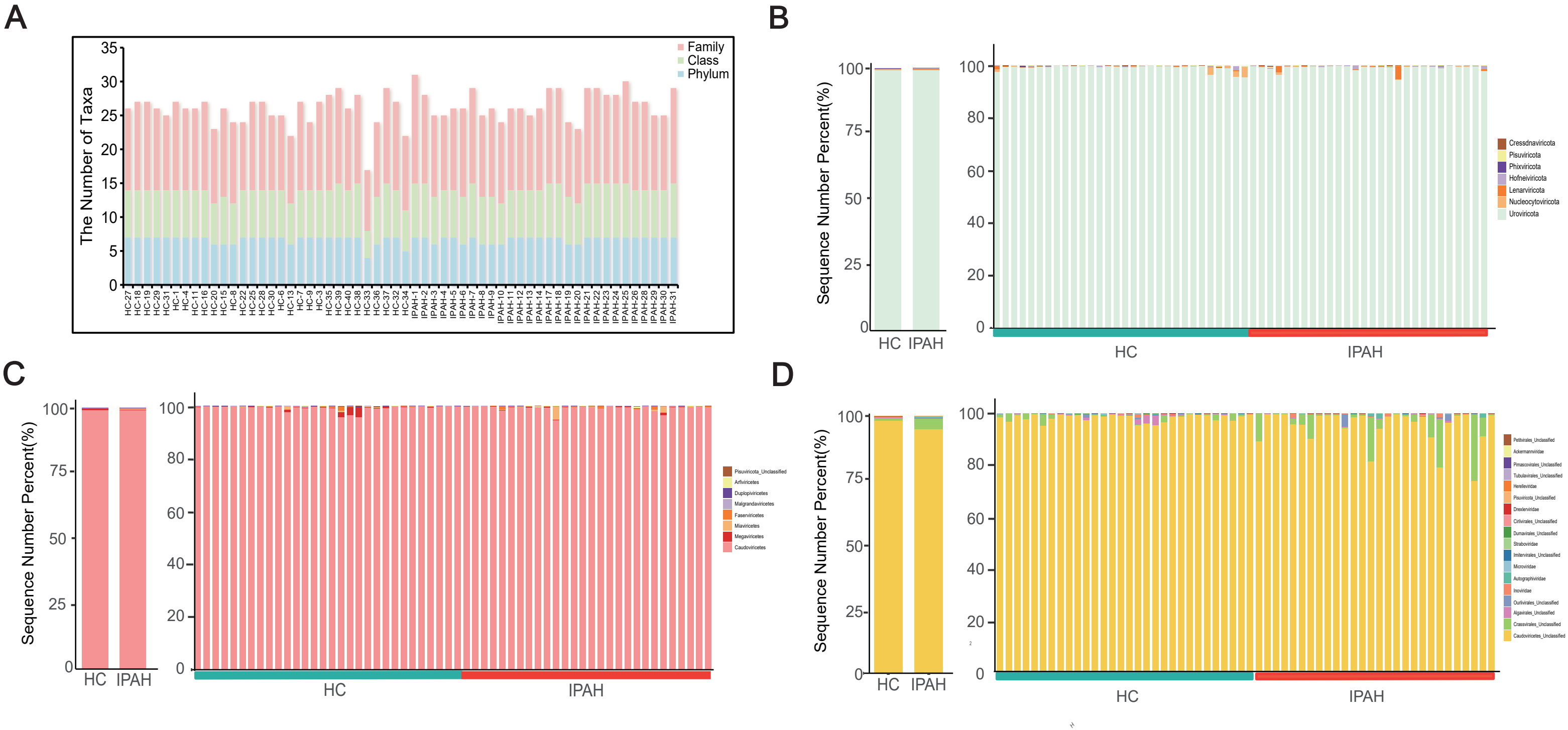

Figure S1 Gut virome profiles of HCs and IPAH patients. (A) Number of viral phyla, classes, and families identified per fecal sample in HCs and IPAH patients. (B) Viral composition at the phylum (B), class (C), and family (D) levels across each group and per sample.

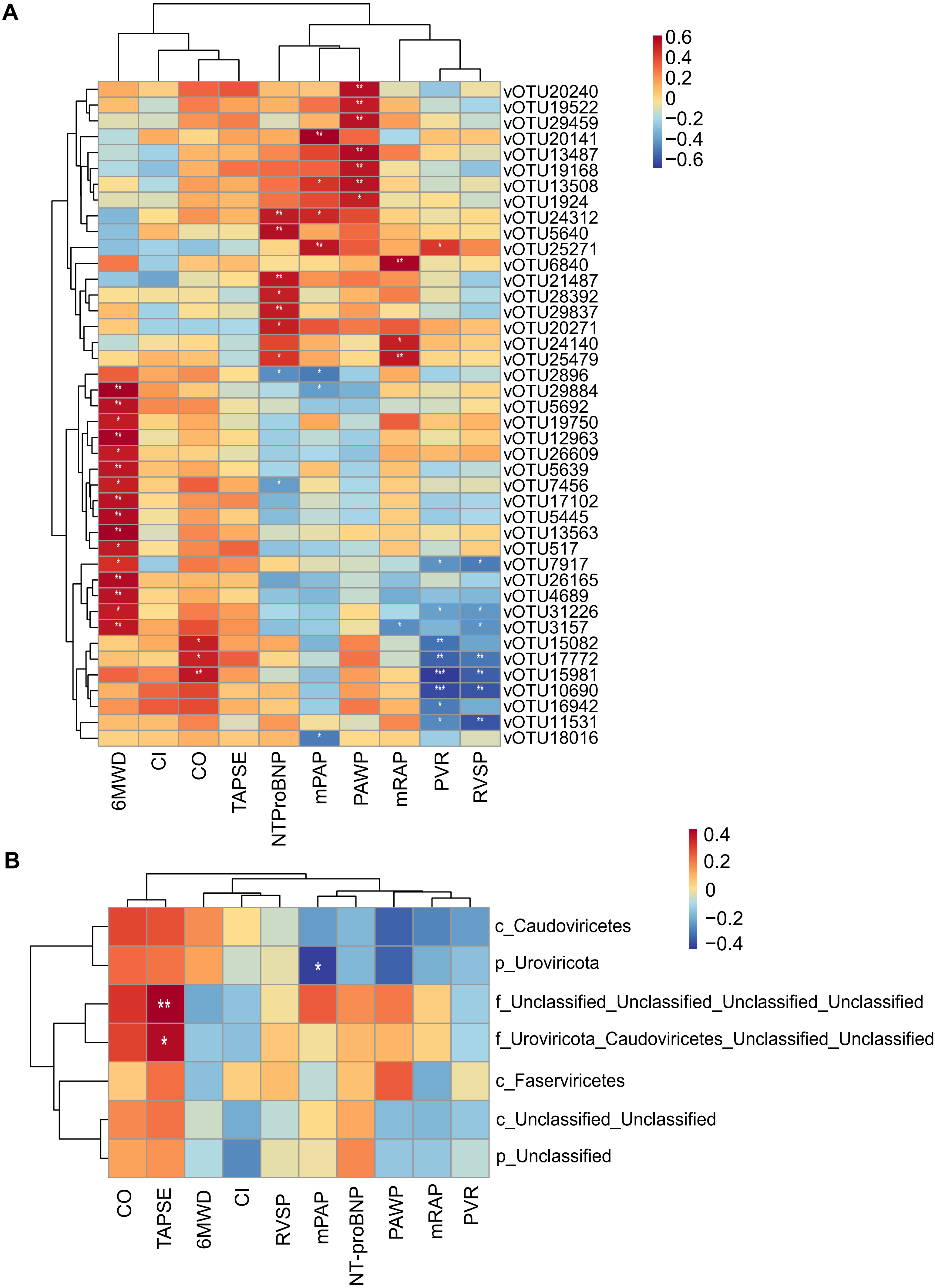

Figure S2. Correlations between differential viruses (vOTUs and family level) and major IPAH clinical indicators. (A, B) Spearman ’ s correlations between differential viruses at the vOTU (A) and viral family (B) levels and major IPAH clinical indicators; *$p < 0.05$; **$p < 0.01$. CI: cardiac index; CO: cardiac output; PVR: pulmonary vascular resistance; PAWP: pulmonary artery wedge pressure; RVSP: right ventricular systolic pressure; mRAP: mean right atrial pressure; NT-proBNP: N-terminal pro-brain natriuretic peptide; 6MWD: 6-minute walk distance.

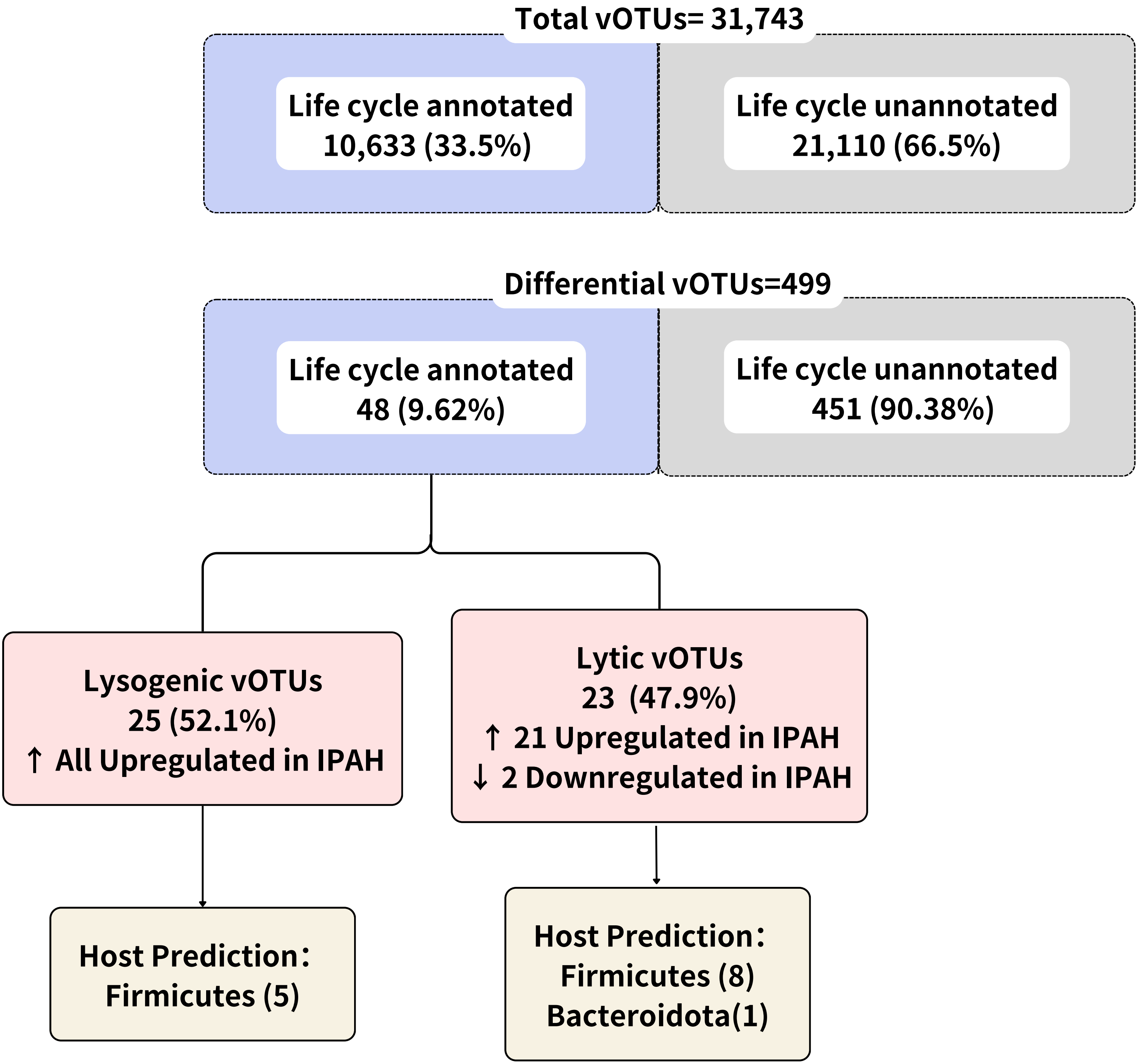

Figure S3. Life cycle characteristics and differential features of vOTUs. Both annotated and unannotated vOTUs are shown.

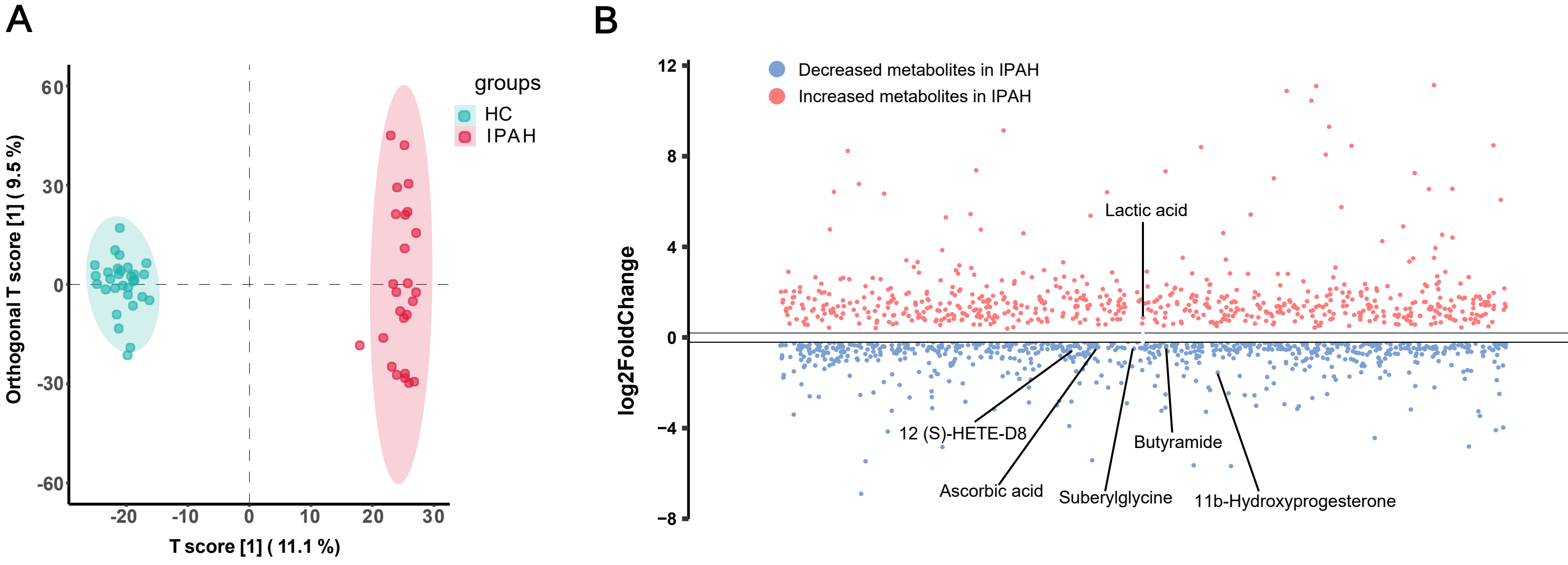

Figure S4. Differential metabolite profiles between IPAH patients and HCs. (A) OPLS-DA score plot showing clear separation between the IPAH and HC groups. (B) Volcano plot generated with OmicStudio (https://www.omicstudio.cn/tool), highlighting key differential metabolites identified via mediation analysis.

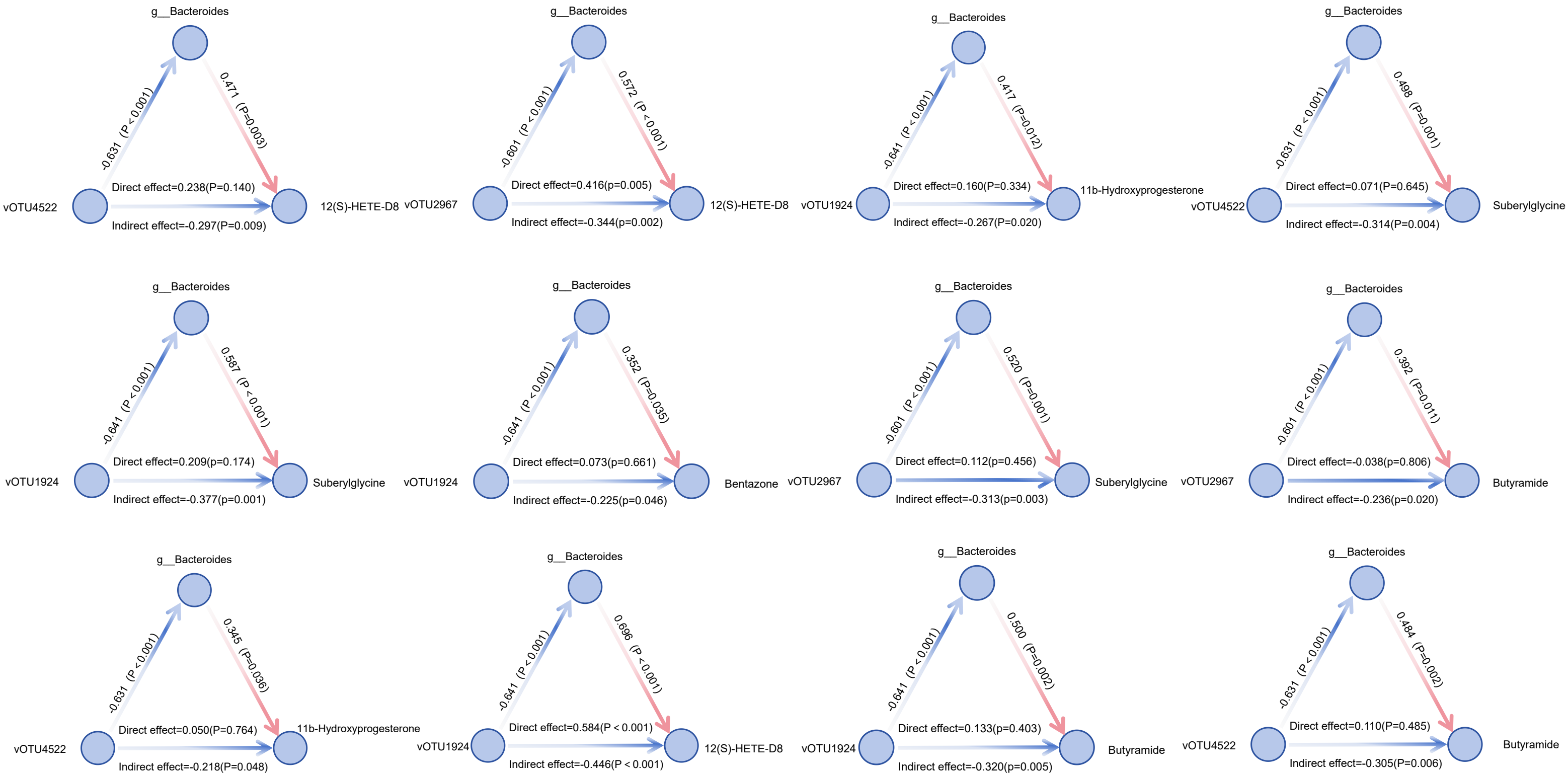

Figure S5. Mediating links among gut viruses, bacteria, and metabolites. Representative mediation pathways inferred among gut vOTUs, gut bacterial taxa, and serum metabolites.
